# 5-*n*-Alkylresorcinol Profiles in Different Cultivars of Einkorn, Emmer, Spelt, Common Wheat, and Tritordeum

**DOI:** 10.1021/acs.jafc.1c05451

**Published:** 2021-11-18

**Authors:** Clara Pedrazzani, Francesca Vanara, Dhaka Ram Bhandari, Renato Bruni, Bernhard Spengler, Massimo Blandino, Laura Righetti

**Affiliations:** † Department of Food and Drug, University of Parma, Parco Area delle Scienze 17/A, Parma 43124, Italy; ‡ Department of Agricultural, Forest and Food Sciences, University of Torino, Largo Paolo Braccini, 2, Grugliasco 10095, Italy; § Institute of Inorganic and Analytical Chemistry, Justus Liebig University Giessen, Heinrich-Buff-Ring 17, Giessen 35392, Germany

**Keywords:** alkylresorcinols, *Triticum* spp., x *tritordeum martinii*, ion mobility mass spectrometry, mass spectrometry imaging, bioactives

## Abstract

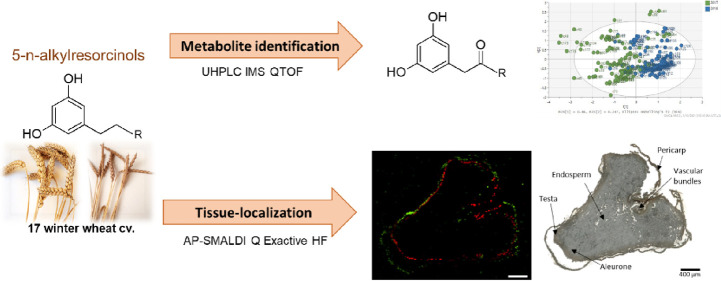

5-*n*-Alkylresorcinols (AR) are bioactive compounds found in the edible parts of many cereals. Here, saturated and unsaturated homologues, including the oxidized forms 5-(2′-oxo) AR and their plant metabolites, were profiled by ultrahigh-performance liquid chromatography–ion mobility separation–high-resolution mass spectrometry in 18 cultivars of einkorn, emmer, spelt, common wheat, and tritordeum, cultivated in two consecutive years under uniform agronomic conditions. The average content of AR ranged between 672.5 ± 129.8 and 1408.9 ± 528.0 mg/kg, exceeding 2380 mg/kg in some samples and highlighting a superior content in tritordeum and in modern cultivars with respect to old wheat genotypes. By evaluating the effect of environmental and agronomic factors on the different variables, the harvest year resulted to be always significant, while location and variety influenced AR abundance only for some homologues. Furthermore, the spatial distribution of AR was investigated by mass spectrometry imaging using transversal cross sections of wheat kernels. Our results show that AR homologues are mainly localized in the testa and in the outer pericarp of wheat kernels.

## Introduction

5-*n*-Alkylresorcinols (AR) are amphiphilic molecules characterized by a polar, resorcinol ring linked to an odd numbered alkyl chain, which at least in cereals generally ranges from 15 to 25 carbon atoms.^1^ In fact, from a nutritional standpoint, their abundance is relevant in some cereals and pseudocereals, and their biological role both *in planta* and in human nutrition^2−4^ has recently garnered renewed interest from breeding and food science; after that, initial attention was focused mainly on the antinutritional role.^1,5^ Indeed, due to their structure, AR may interact with biological membranes inducing multiple biological activities, including antifungal properties^6^ and antimutagenic activity.^7^ Their accumulation in the outer cuticle of the testa and in the inner cuticle of the pericarp of cereals has been linked also to a protective role against plant pathogens.^6^ Furthermore, being present in high amounts in most of the whole grain cereals^1^ commonly used for food, there is growing evidence suggesting a contribution of AR in decreasing the risk of colorectal cancer,^8,9^ as selective biomarkers of whole grain consumption and also as a marker for cereal authenticity.^10^

The main saturated homologues of AR are found in wheat, rye, barley, triticale grains,^1^ and quinoa seeds,^11^ but among food crops, their distribution is uneven, highlighting the relevance of reliable, detailed profiling. Homologues ranging from C17 to C25 are in fact present in high concentrations in wheat, rye, and quinoa whole grains, less abundant in barley, but are not produced in mature, ungerminated maize and rice grains,^12^ while no information is at present available for tritordeum. Among different *Triticum* spp., the total AR content may vary between hexaploid, tetraploid, and diploid species, suggesting a remarkable heritability of their content and an important role of the genetic background on their accumulation.^13,14^ Furthermore, as already reported for other phenolic compounds, AR composition is strongly affected by environmental and agronomic conditions.^13^ For instance, their content may range from 250 to 1400 and from 200 to 950 μg/g dry matter in whole grains rye and wheat, respectively.^5^ Hexaploid tritordeum, in particular, is an amphidiploid cereal derived from the cross between wild barley (*Hordeum chilense* Roem. et Schultz.) and cultivated durum wheat (*Triticum turgidum* (*T. turgidum*) L. ssp. *durum* Desf.), whose use in food processing is increasing.^15−17^ To our knowledge, the occurrence and profile of AR in tritordeum (x *tritordeum martinii* A. Pujadas, nothosp. nov.) (AABBHchHch) have not been previously reported in the literature.^18^

On the other hand, due to the renewed interest in these plant secondary metabolites and to the availability of more potent analytical approaches, more detailed investigations unraveled the complexity of AR accumulation in plants. Several branched-chain and methyl-alkylresorcinol homologues have been recently identified in quinoa,^11^ while 5-(2-oxo)alkylresorcinols were reported for the first time in wheat^19^ and rye.^20^ Furthermore, conjugated forms of these phenolic lipids (i.e., glucosilated metabolites) were reported in *Cybianthus magnus*(21) and *Grevillea robusta.*(22) While the composition of the most common AR is available for many cultivars of einkorn, emmer, spelt, and common wheat, limited data are available regarding the presence of recently described homologues. Moreover, the comparison of plant material obtained from different geographical locations and under distinct agronomic practices may lead to scattered and unreliable results. In particular, recent reports confirmed that different stresses experienced by rye in separate growing seasons may lead to consistent variability in the AR content, thus highlighting the need for evaluations taking into account multiple harvests in the same location.^23^

Therefore, in this work, we aimed at characterizing the AR homologue profiles in different cultivars of einkorn (*Triticum monococcum* (*T. monococcum*) spp. *monococcum*), emmer (*T. turgidum* spp. *dicoccum*), spelt (*Triticum aestivum* (*T. aestivum*) spp. *spelta*), common wheat (*Triticum aestivum* (*T. aestivum*) spp. *aestivum*), and tritordeum, establishing the genetic and environmental contribution to their variability. In addition to AR quantification, advanced analytical techniques were applied to investigate the formation of AR metabolites and to finely locate them within the cereal kernel.

## Materials and Methods

### Chemicals and Reagents

5-Nonadecyl-resorcinol, 5-heneicosyl-resorcinol, 5-tricosyl-resorcinol, 5-heptadecylresorcinol (10 mg powder), 2,5-dihydroxybenzoic acid (DHB), and trifluoroacetic acid (TFA) were purchased from Sigma-Aldrich (Steinheim). Gelatin used for embedding was obtained from VWR International (Darmstadt, Germany). Glass microscope slides (ground edges, super frost) were obtained from R. Langenbrinck (Emmendingen, Germany). LC–MS-grade methanol, ethyl acetate, and 2-propanol were purchased from Scharlab Italia Srl (Milan, Italy); bidistilled water was obtained using a Milli-Q system (Millipore, Bedford, MA, USA). MS-grade ammonium formate and formic acid from Fisher Chemical (Thermo Fisher Scientific, Inc., San Jose, CA, USA) were also used.

### Sampling Plan

Eighteen winter varieties of *Triticum* spp. and tritordeum were collected, including diploid, tetraploid, and hexaploid species (Table 1). As far as common wheat is concerned, old genotypes (year of release before 1985)^24^ and pigmented variety rich in carotenoids or anthocyanins^25^ were compared to several conventional modern cultivars (see Table 1 for details). Each selected cultivar was simultaneously cultivated over two growing seasons (2016–2017 and 2017–2018) in two different locations in the Northwest Italian plains, namely, Carmagnola (44°50′ N, 7°40′ E; elevation of 245 m, deep fertile silty-loam soil) and Cigliano (45°18′ N, 8°01′ E; elevation of 237 m, in shallow loam soil), with a lower cation-exchange capacity and organic matter content. Each plot had a 7 × 1.5 m size.

**Table 1:** Cultivars of Einkorn, Emmer, Spelt, Common Wheat, and Tritordeum Considered for This Study

species		ploidy level^a^	type	cultivar	seed company	year of release
einkorn	*T*. *monococcum* spp. *monococcum*	diploid (AA)		Monlis	Prometeo, Urbino, Italy	2006
emmer	*T*. *turgidum* spp. *dicoccum*	tetraploid (AABB)		Luni	S.I.S., San Lazzaro di Savena, Italy	2002
				Giovanni Paolo	Apsovsementi, Voghera, Italy	2008
spelt	*T*. *aestivum* spp. *spelta*	hexaploid (AABBDD)		BC Vigor	Bc Institute, Zagreb, Croatia	2012
				Rossella	Apsovsementi, Voghera, Italy	2016
common wheat	*T*. *aestivum* spp. *aestivum*	hexaploid (AABBDD)	old^b^	Andriolo	Italian local landrace	from XIX° century
				Gentilrosso	Italian local landrace	from XIX° century
				Frassineto	Italian local landrace	1922
				Verna	Italian local landrace	1953
			modern	Bologna	S.I.S., San Lazzaro di Savena, Italy	2002
				Aubusson	Limagrain Italia, Fidenza, Italy	2003
				Solehio	Agroalimentare Sud Spa, Melfi, Italy	2008
				Arabia	Apsovsementi, Voghera, Italy	2009
			pigmented^c^	Bonavita (yellow-grained)	Osivo a. s., Zvolen, Slovakia	2011
				Rosso (purple-grained)	Saatbau, Leonding, Austria	2011
				Skorpion (blue-grained)	Agricultural Research Institute, Kromeriz, Czech Republic	2013
tritordeum	X *tritordeum martinii*	hexaploid (AABBHchHch)		Aucan	Agrasys S.L., Barcelona, Spain	2011
				Bulel	Agrasys S.L., Barcelona, Spain	2011

a Ploidy level = number of sets of chromosomes.

b Andriolo, Gentilrosso, Frassineto, and Verna were historically cultivated common wheat cultivars (before 1985), while all the other common wheat, einkorn, emmer, spelt, and tritordeum cultivars are modern genotypes, released after 2000.

c Cultivars with a high content of carotenoids (Bonavita) and anthocyanins (Rosso and Skorpion), responsible for the yellow and blue-purple hue of kernels, respectively, in comparison to the conventional white- or red-grained wheat varieties.

The same agronomic technique was adopted for all cultivars (see the Supporting information).

The plots were harvested using a Walter Wintersteiger cereal plot combine harvester, and the grain yield results were adjusted to a 13% moisture content. After harvesting, the husks of einkorn, emmer, and spelt were removed through a laboratory dehusking machine (FC2K Otake, Dellavalle Srl, Mezzomerico, Italy). Aliquots of 2 kg of grains were taken from each plot to determine the test weight (TW), the thousand-kernel weight (TKW), and the grain moisture content, using a GAC 2000 grain analyzer (Dickey-John, Auburn, IL, USA). The TKW was determined on two 100-kernel sets for each sample (only whole seeds without husks were considered) using an electronic balance. Kernels of each plot were milled through a laboratory centrifugal mill (Model ZM-100, Retsch, Haan, Germany) equipped with a 1 mm sieve and homogenized. Prior to chemical analyses, all the samples were ground to a fine powder (particle size of <300 μm) with a Cyclotec 1093 sample mill (Foss, Padova, Italy) and stored for 2 weeks at −25 °C until the beginning of the analyses.

### AR Extraction

The process was optimized considering (i) the solvent-to-solid ratio, where 1 g of the sample was extracted with 20 mL or 30 mL of ethyl acetate; (ii) extraction cycle duration, where samples were extracted by shaking for 60, 90, and 120 min; and (iii) extraction repetition, where the procedure was repeated up to 3 times until exhaustion.

One gram of ground cereals was stirred for 60 min at 240 strokes/min with 20 mL of ethyl acetate and then centrifuged for 10 min at 14,000 rpm. The supernatant (1000 μL) was dried under nitrogen flow. After two repetitions, the supernatants were pooled, reconstructed into 1 mL of mobile phase B, and injected into the UHPLC–TWIMS–QTOF.

### UHPLC–TWIMS–QTOF Analysis

An ACQUITY I-Class UPLC separation system coupled to a Vion IMS QTOF mass spectrometer (Waters, Wilmslow, UK) equipped with an electrospray ionization (ESI) interface was employed for AR profiling. Samples were injected (1 μL) and chromatographically separated using a reversed-phase C18 BEH ACQUITY column (2.1 × 100 mm, 1.7 μm particle size) (Waters, Milford, MA, USA). Gradient elution was performed as previously reported,^26^ by using 5 mM ammonium formate in Milli-Q water/methanol (95:5, v/v) (solvent A) and 5 mM ammonium formate in isopropanol/methanol/Milli-Q water (65:30:5, v/v) (solvent B) both acidified with 0.1% formic acid. The following multistep elution gradient was used: 0.0 min (10% solvent B; 0.40 mL/min) to 1.0 min (50% solvent B; 0.40 mL/min), subsequently 1–5 min (80% solvent B; 0.40 mL/min), and 11.0 min (100% solvent B; 0.50 mL/min). After a 4.5 min isocratic step, the system was re-equilibrated to initial conditions for 2.5 min (10% solvent B; 0.4 mL/min). Samples were permanently kept at 10 °C.

Mass spectrometry data were collected in negative electrospray mode over the mass range of *m*/*z* 100–1000. Source settings were maintained using a capillary voltage of 2.5 kV, a source temperature of 120 °C, a desolvation temperature of 500 °C, and a desolvation gas flow of 1000 L/h. A TOF analyzer was operated in sensitivity mode, and data were acquired using HDMSE, which is a data-independent approach (DIA) coupled with ion mobility. The optimized ion mobility settings included a nitrogen flow rate of 90 mL/min (3.2 mbar), a wave velocity of 650 m/s, and a wave height of 40 V. The device within the Vion was calibrated using a Major Mix IMS calibration kit (Waters, Wilmslow, UK) to allow for CCS values to be determined in nitrogen. The calibration covered the CCS range from 130 to 306 Å^2^. The TOF was also calibrated prior to data acquisition and covered the mass range from *m*/*z* 151 to 1013. TOF and CCS calibrations were performed for both positive- and negative-ion mode. Data acquisition was conducted using UNIFI 1.8 (Waters, Wilmslow, UK).

Alkylresorcinol identification was performed by comparison of retention time, fragmentation patterns, and collision cross sections with the standard collection in our UNIFI library, created by running a mix of standards with the same analytical method. Quantification of target analytes was performed using external standard calibration (range of 0.1–25 mg/kg). Quantification of unsaturated AR was based on the relative response of an alkylresorcinol with the same chain length due to the lack of analytical standards. AR values were reported on a dry matter (DM) basis. The main validation parameters are briefly summarized in the Supporting Information (Table S1).

### Statistical Analysis

Statistical analyses were performed using IBM SPSS v.23.0 (SPSS Italia, Bologna, Italy). Data were analyzed by ANOVA followed by Tukey’s post hoc test (α = 0.05). Principal component analysis was constructed with log-normalized and pareto-scaled data using SIMCA V.16.0.2 (Umetrics, Malmö, Sweden).

### AP-SMALDI MSI Sample Preparation and Analysis

Sample preparation for atmospheric-pressure scanning microprobe matrix-assisted laser desorption/ionization mass spectrometry imaging (AP-SMALDI MSI) was performed following the protocol previously optimized.^27^ Briefly, samples were embedded in 2% gelatin, and then, 20 μm-thick sections were cut from the middle of each grain at −20 °C using a cryomicrotome (HM525 cryostat, Thermo Fisher Scientific, Dreiech, Germany). To obtain uniform sections, due to the fragility of the seed, adhesive tape kept over the trimmed sample during cryosectioning was used. The sections were transferred to a glass slide and kept at −80 °C until the day of the analysis. Before matrix application, optical images of the sections were captured using a digital microscope VHX-5000 (Keyence GmbH, Neu-Isenburg, Germany). DHB (10 mg mL^–1^) in acetonitrile (70:30, v/v, 0.1% TFA) was sprayed with a pneumatic sprayer (SMALDIPrep, TransMIT GmbH, Giessen, Germany)^28^ to ensure uniform coating of tissue sections with the microcrystalline matrix. The size and uniformity of the deposited crystals were checked prior to AP-SMALDI MSI experiments.

This combination of a matrix and a solvent was chosen because it gives rise to the highest signal intensities for alkylresorcinol standards, following the protocol previously optimized for urushiol MALDI ionization.^29^ The dried-droplet method was used to assess different matrices, by mixing 1 μL of an alkylresorcinol standard with 1 μL of matrix solution and spotting 0.5 μL onto an 80-well stainless-steel plate.

Imaging experiments of wheat seed sections were performed using a high-spatial-resolution (≥5 μm step size) AP-SMALDI MSI ion source (AP-SMALDI5 AF, TransMIT GmbH, Giessen, Germany) coupled to a Q Exactive HF orbital trapping mass spectrometer (Thermo Fisher Scientific GmbH, Bremen, Germany).

The smallest laser beam focus resulted in an ablation spot diameter of 5 μm.^30^ For the experiments described below, a scanning step size of 20 μm was set. The mass spectrometer was operated in positive-ion mode. The following parameters were set: scan range, *m*/*z* 250–1000; target voltage, +3 kV; capillary temperature, 250 °C; automatic gain control (AGC) was disabled; cycle time for one pixel, 1 s. Internal mass calibration was performed using known matrix ion signals as lock mass values (*m*/*z* 716.12462), providing a mass accuracy of better than 2 ppm root-mean-square error over the entire measurement.

Ion images of selected *m*/*z* values were generated using the MIRION software package^31^ with a bin width of Δ(*m*/*z*)/(*m*/*z*) = ±5 ppm. MS images were normalized to the highest intensity of each ion species. The METASPACE platform (https://metaspace2020.eu/) was employed for metabolite annotation of MSI data, selecting Metlin and LIPID MAPS as databases.

## Results and Discussion

### Sample Preparation Method Development and Optimization

Extraction recovery is a key step in the characterization of bioactive compounds in plants. Here, we optimized the winter cereal sample preparation to obtain a complete recovery of AR.

Starting from the solvent/matrix ratio, a greater volume (1 g with 30 mL) did not provide a higher AR recovery or other advantages if compared to 20 mL. The extraction efficiency did not increase with time (60, 90, and 120 min), suggesting that the equilibrium of the solute inside and outside the plant matrix was reached after 60 min. On the other hand, repetition of the extraction process with a fresh solvent increased the content of AR, suggesting a saturation of the extraction solvent during the first cycle. The optimized sample preparation finally included two extraction cycles of 60 min using 20 mL of ethyl acetate. The first cycle extracted 92.7% (±1.05) of the total AR, while the second cycle extracted 7.3% (±1.14). The procedure was repeated up to 3 times, but in the third extraction, no AR were detected, indicating that the matrix was exhausted after 2 cycles. Compared to previously developed extraction procedures, a shorter extraction time^14^ and no derivatization steps^1^ are required.

### AR Contents in *Triticum* spp.

The presence of AR homologues (AR 17:0–AR 25:0) in selected grains was measured by UHPLC–IMS–HRMS. The average content of AR ranged between 672.5 ± 129.8 and 1408.9 ± 528.0 mg/kg of dry matter (DM) among different cultivars of *Triticum* spp. and tritordeum (Table 2). These data are slightly exceeding previous reports in which the average AR contents of spelt, emmer, and einkorn were in the 580–820 mg/kg DM range.^32^ The qualitative profile also varied strongly, and the dominant AR homologues were AR 19:0, AR 21:0, and AR 23:0. Seven different AR homologues (5 saturated and 2 unsaturated) were identified in winter cereals, which agreed with previous reports.^33^ In particular, the predominant homologue in all five species was AR 21:0, representing almost half of the total AR content, with a percentage ranging from 48.1 (tritordeum) to 58.3% (emmer). AR 19:0 was the second most abundant homologue in the hexaploid species *T. aestivum* spp. *aestivum* (25.3%) and spp. *spelta* (26.1%) followed by AR 23:0, AR 19:1, AR 17:0, AR 21:1, and AR 25:0 (see Figure S1 of the Supporting Information). In contrast, tritordeum, emmer, and einkorn contained higher portions of AR 23:0, that is, 32.9, 28.4, and 28.3%, respectively, followed by the homologues AR 19:0, AR 25:0, AR 21:1, and AR 19:1 (Table 2). This trend was in agreement with previously reported data.^1,33,34^

**Table 2:** Total Alkylresorcinol (AR) Content and Relative Homologue Composition from Cultivars of Einkorn, Emmer, Spelt, Common Wheat, and Tritordeum^a^

			total AR (mg/kg, DM)	AR homologue composition (%)							AR ratio	
species		variety	mean ± SD	17:0	19:0	21:0	23:0	19:1	21:1	25:0	17:0/21:0	21:0/23:0
einkorn		Monlis	770.0 ± 251.9	<LCL	15.6	53.2	28.3	0.9	1.4	1.0	<LCL	1.90 ± 0.25ab
emmer		Luni	877.8 ± 111.8	2.0	11.2	56.7	28.3	1.3	1.3	1.7	0.04 ± 0.00	2.04 ± 0.38
		Giovanni Paolo	808.9 ± 92.7	<LCL	7.2	60.0	28.5	1.4	1.6	1.8	<LCL	2.13 ± 0.25
			843.3 ± 106.4	2.0	9.2	58.3	28.4	1.3	1.4	1.7	0.04 ± 0.00	2.08 ± 0.32b
												
spelta		BC Vigor	939.0 ± 96.5	2.5	27.7	54.3	10.7	3.0	1.3	1.2	0.05 ± 0.02	5.18 ± 0.89
		Rossella	672.5 ± 129.8	<LCL	24.3	53.8	13.0	5.1	2.7	1.7	<LCL	4.18 ± 0.69
			817.9 ± 174.7	2.5	26.1	54.1	11.7	4.0	2.0	1.4	0.05 ± 0.02	4.73 ± 0.91d
												
common wheat	old	Andriolo	772.4 ± 89.5	4.2	23.4	52.1	16.2	2.9	1.7	2.2	0.08 ± 0.01	3.26 ± 0.40
		Gentilrosso	719.0 ± 154.3	5.9	23.9	48	13.1	4.9	3.1	3.1	0.12 ± 0.04	3.75 ± 0.72
		Frassineto	774.7 ± 163.0	4.9	26.6	47.3	12.5	5.1	2.7	3.1	0.10 ± 0.04	3.81 ± 0.50
		Verna	758.0 ± 146.1	4.2	26.6	47.0	13.0	5.4	2.6	3.0	0.09 ± 0.02	3.64 ± 0.32
	modern	Bologna	1408.9 ± 528.0	2.8	25.8	50.8	13.5	3.2	3.0	1.6	0.06 ± 0.02	3.77 ± 0.30
		Aubusson	1307.7 ± 391.3	3.0	25.6	51.7	14.5	2.7	1.9	1.4	0.06 ± 0.01	3.72 ± 0.84
		Solehio	758.1 ± 148.7	5.1	28.6	44.9	11.5	5.3	3.4	2.8	0.12 ± 0.05	3.95 ± 0.68
		Arabia	946.0 ± 239.9	3.8	28.0	51.9	10.5	3.1	3.1	1.5	0.07 ± 0.01	4.95 ± 0.49
	pigmented	Bonavita	1239.8 ± 368.3	2.6	18.6	56.5	15.8	3.4	2.9	1.9	0.05 ± 0.02	3.58 ± 0.26
		Rosso	979.3 ± 231.5	4.4	29.8	46.5	11.3	4.3	2.1	2.6	0.10 ± 0.02	4.18 ± 0.62
		Skorpion	857.6 ± 229.0	4.7	22.0	50.3	14.5	4.4	3.5	2.9	0.10 ± 0.04	3.50 ± 0.59
			956.5 ± 356.1	4.1	25.3	49.7	13.3	4.1	2.7	2.4	0.09 ± 0.04	3.83 ± 0.68c
												
tritordeum		Aucan	978.0 ± 226.54	3.1	11.4	46.8	34.9	1.4	1.8	4.2	0.06 ± 0.01	1.35 ± 0.13
		Bulel	1172.0 ± 581.12	<LCL	11.9	49.3	31.0	2.6	2.8	3.7	<LCL	1.65 ± 0.29
			1075.0 ± 442.6	3.1	11.7	48.1	32.9	1.9	2.2	3.9	0.06 ± 0.01	1.50 ± 0.27a

a Data are average of 2 sites in Northwest Italy and 2 years (harvest in 2017 and 2018). The LCL (lowest calibration level) for AR 17:0 is 100 μg/kg. Means of species followed by different letters are significantly different (*p* < 0.05), according to the Tukey’s post hoc test.

Recent studies involving different *Triticum* species indicated that the accumulation of AR is influenced both by the genetic background and the environmental factors.^1,35,36^ Therefore, MANOVA was carried out on AR homologues, considering their accumulation over the growing locations, varieties, and harvesting years. By evaluating the effect of each factor on the different variables, the year resulted to be always significant (*p* < 0.01) while location and variety only for some homologues as shown in Table 3.

**Table 3:** Results of the Multivariate Analysis of Variance (MANOVA) for Principal Homologues toward the Location, Year, and Variety (*p* < 0.05) on Alkylresorcinol Profiles

effect	AR 17:0	AR 19:0	AR 21:0	AR 23:0	AR 25:0
location	0.003	0.035	0.130	0.221	0.025
year	<0.000	<0.000	0.003	<0.000	<0.000
variety	0.806	<0.000	<0.000	<0.000	0.149

**Figure 1:**
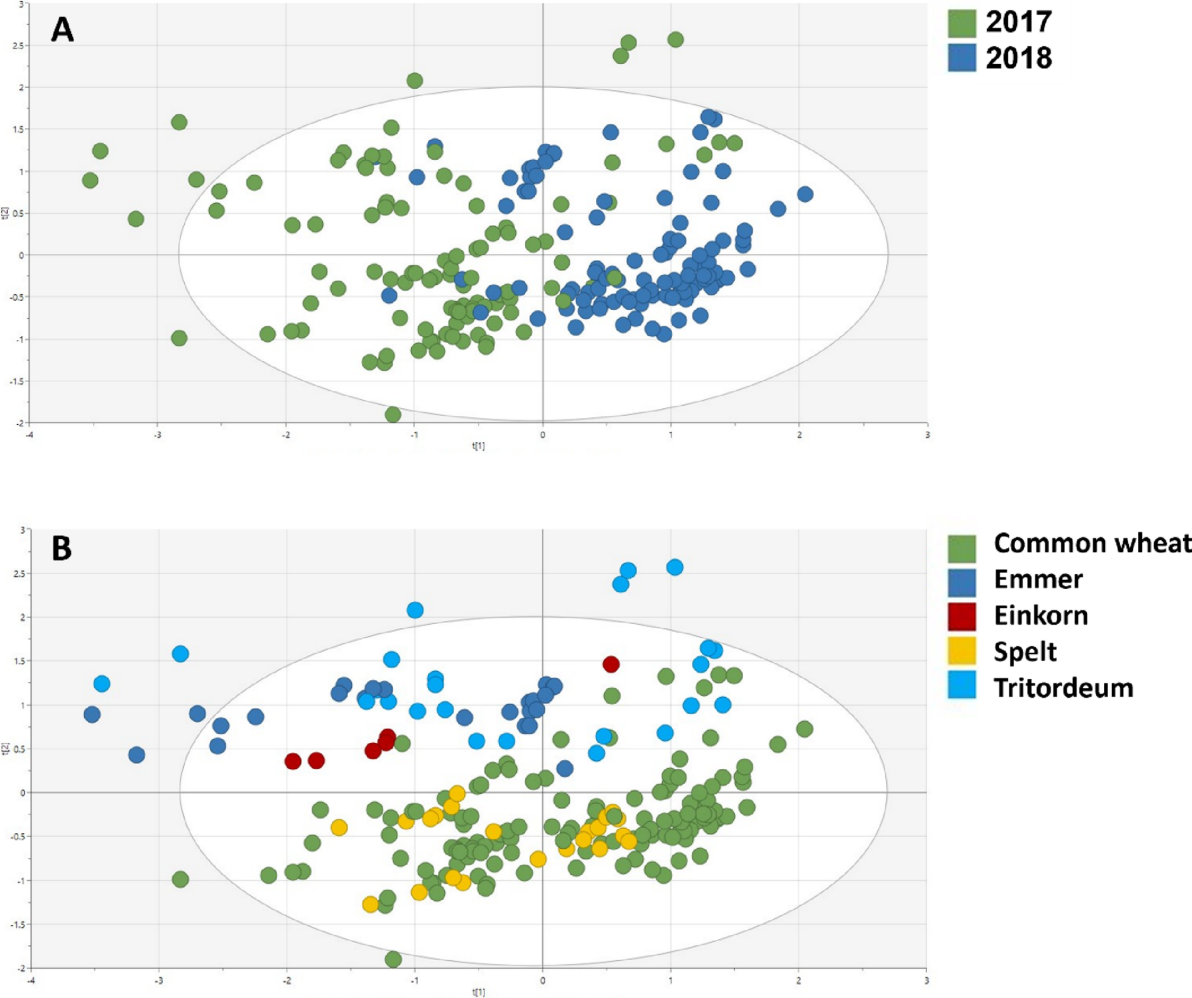
PCA score plot built with the two principal components (PC1 and PC2) (*R*^2^*Y* = 0.708; *Q*^2^ = 0.493) and colored according to (A) harvesting years and (B) genetic species.

Similarly, the PCA in Figure 1 shows both the environmental and genetic influences on the total AR contents. Indeed, the first PC clustered the samples according to the harvesting years (Figure 1A), while the second PC (Figure 1B) separated the different species. No separation was observed according to the growing locations. Regarding the harvesting year, a higher AR content was detected in 2018 compared to 2017. This trend might be explained considering the meteorological conditions registered during the growing seasons and their consequences to kernel traits. In 2017–2018, more intense precipitation was recorded from April to May, from the beginning of heading to the soft dough stage, under warmer temperatures, while 2016–2017 was characterized by drier meteorological trends (see Table S2 of the Supporting Information). As a consequence, in both sites, the severity of foliar (Septoria leaf blotch) and head (Fusarium head blight) disease in 2017–2018 was higher, leading to a quicker senescence process and a clearly lower test weight (TW) and thousand-kernel weight (TKW). On average, grain yields were 4.2 and 3.3 t ha^–1^ in 2017 and 2018, respectively (see Table S3 of the Supporting Information), as a consequence of the lower TW (72.1 vs 67.2 kg hl^–1^) and TKW (45.3 vs 38.7 g). A low starch accumulation in the grain during ripening in 2018 may have determined a lower dilution of the AR contents, concentrated mainly in the outer layer.^37^

When considering data from all varieties of single species across the two locations and the two years, the hexaploid species tritordeum (1075.0 ± 442.6 mg kg^–1^ DM) and common wheat (956.5 ± 356.1 mg kg^–1^ DM) contained the highest AR concentration followed by the tetraploid emmer (843.3 ± 106.4 mg kg^–1^ DM) and hexaploid spelt (817.9 ± 174.7 mg kg^–1^ DM). Diploid einkorn (770.0 ± 251.9 mg kg^–1^ DM) exhibited the lowest AR concentrations (Table 2). While *T. monococcum* spp. *monococcum* and *T. turgidum* spp. *dicoccum* are often supposed to have higher contents of alkylresorcinols,^33^ our study revealed significantly higher AR contents in common wheat compared to einkorn and emmer, in agreement with our previous study^14^ and the results reported by Ross.^38^

**Figure 2:**
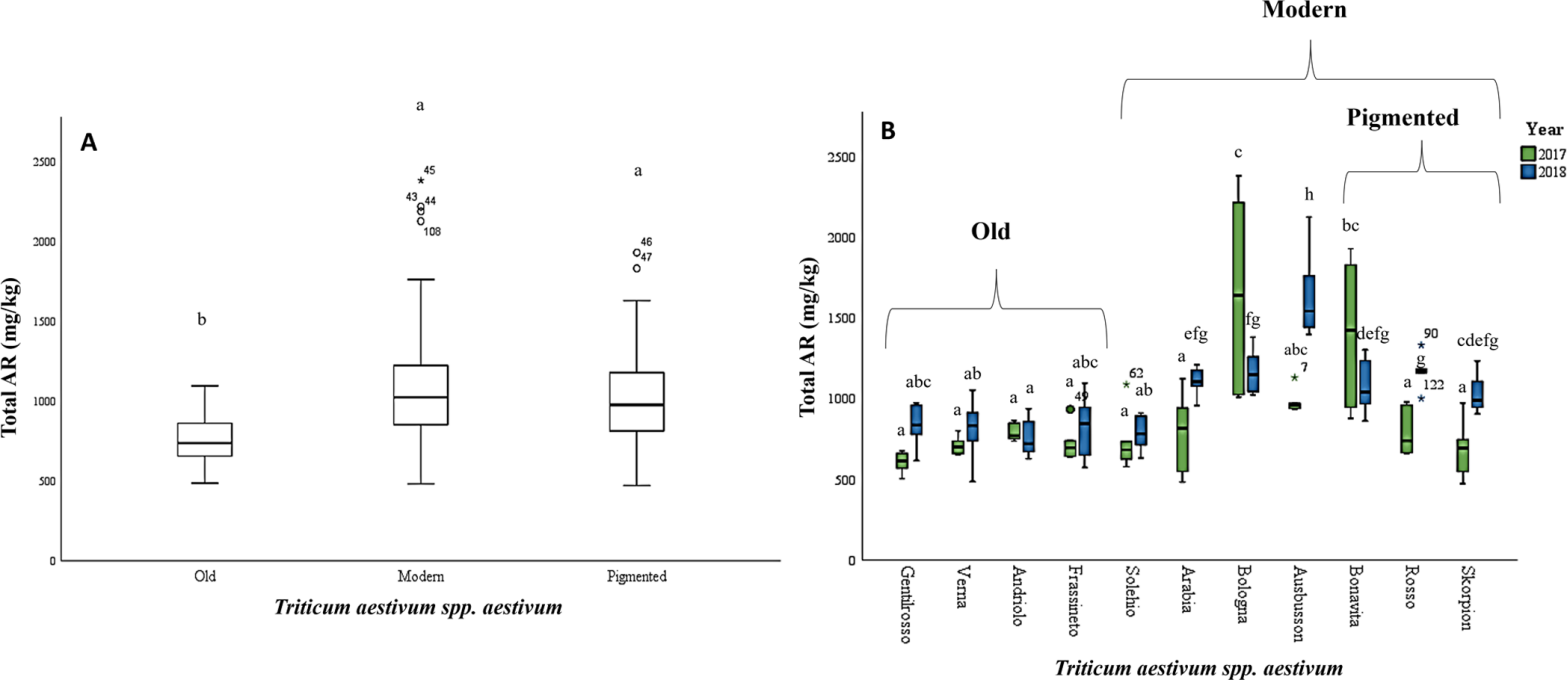
Genetic and environmental variability of AR in common wheat (*T. aestivum* spp. *aestivum*). (A) Box plot of total AR in common wheat grouped into old, modern, and pigmented varieties. Values with different letters differ significantly by Tukey’s post hoc test (*p* < 0.05). (B) Box plot of total AR in common wheat in 2017 (green series) and 2018 (blue series) harvesting years. Values with different letters differ significantly by Tukey’s post hoc test (*p* < 0.05).

Focusing on common wheat, 11 cultivars were analyzed including old, modern, and pigmented varieties (see Table 1). The AR content was significantly higher in modern cultivars than in old wheat genotypes. In particular, the highest AR content was observed for Bologna (1408.9 ± 528.0 mg kg^–1^ DM) and Aubusson (1307.7 ± 391.3 mg kg^–1^ DM) modern wheats (see Figure 2A). On the other hand, old varieties including Gentilrosso (719.0 ± 154.3 mg kg^–1^ DM) and Andriolo (772.4 ± 89.5 mg kg^–1^ DM) reported the lowest AR content. Previous reports on modern and old wheat cultivars were mainly focused on the comparison of the total phenolic content,^39^ while no data on AR have been reported so far. The higher accumulation of AR in modern genotypes may be explained considering the differences in kernel size among genotypes. Indeed, modern cultivars (i.e., Bologna, Bonavita, and Aubusson) are characterized by a small kernel size and a higher pericarp/endosperm ratio compared to old genotypes that are considerably larger (see TKW values, Table S3 of the Supporting Information).

The AR contents of pigmented wheats were in line with those of the other modern varieties. These grains are characterized by a higher concentration of carotenoids (cv. Bonavita) or anthocyanins (cv. Rosso and Skorpion),^25^ but no difference in the phenolic lipid was found. This may be explained by the involvement of separate biosynthetic pathways or at least by an independent regulation of alkylresorcinol synthases, as in the case of anthocyanins where both classes share the involvement of type III PKS enzymes.^40^

Regarding the environmental effects, the contents of AR significantly changed (*p* < 0.05) over the two harvesting years only for modern common wheat varieties (conventional as Arabia, Aubusson, and Bologna and pigmented as Bonavita, Rosso, and Skorpion) and not for old ones (Figure 2B). This greater variability could be explained considering that old varieties are less susceptible to the environmental conditions than the modern ones.^41^ In particular, the variation of the TKW in 2018 compared to 2017 was higher in modern (−14%) and pigmented (−20%) than old genotypes (−6%) (see Table S3 of the Supporting Information). Since old wheat cultivars have a lower capacity to respond to production factors with a modification in the yield components and the grain traits (e.g., kernel dimension and density), a greater stability of their composition is expected also within different production situations, as far as the intensity of the cropping system is concerned (e.g., fertilization and disease control treatments).^25^

Furthermore, the ratio of some AR homologues was calculated and discussed (Table 2). In particular, the AR 17:0/AR 21:0 ratio is used as a marker of authenticity to differentiate *Triticum* species.^42^ The ratios for common wheat (0.09 ± 0.04), spelt (0.05 ± 0.02), and emmer (0.04 ± 0.00) were in line with those reported in the literature,^14^ while for einkorn, this evaluation was not possible with AR 17:0 < LOQ. AR 17:0 is known to be present in einkorn,^38^ even if in a small amount, but we could not detect it probably due to its low ionization efficiency obtainable with an ESI source, compared to other sources such as APCI.^43^

The indicator significantly decreased according to hexaploid > tetraploid ≥ diploid. This confirmed the important role of the genetic background on the accumulation of AR in wheat, in agreement with evidence in the literature reporting on the remarkable heritability of the AR content, and the differences in AR profiles between hexaploid, tetraploid, and diploid *Triticum* species.^44^

On the other hand, the ratio AR 21:0/AR 23:0 is considered as an indicator of antifungal activity.^6^ As reported in Table 2, this ratio is strongly related to the ploidy level, with the hexaploid common wheat (3.83 ± 0.68) and spelt (4.73 ± 0.91) showing the highest ratio followed by emmer (2.08 ± 0.32) and einkorn (1.90 ± 0.25). These concentrations are in line with those reported in our previous publication,^14^ suggesting the stability of this indicator despite the great variability of environmental conditions. Indeed, even though the absolute concentration of AR 21:0 and AR 23:0 varied significantly (see Table 3), their ratio remained stable. This will suggest the inclusion of this ratio in cereal breeding programs to obtain new genotypes with increased resistance against fungal ear infections.

### AR Content of Tritordeum

To our knowledge, the profiling of AR in tritordeum has not been previously reported in the literature. Here, we considered two x *tritordeum martinii* varieties, namely, Aucan and Bulel. As shown in Table 2, the total AR contents were found ranging from 679.4 to 2216.3 mg kg^–1^ with a mean content of 1075.0 ± 442.6 mg kg^–1^ DM.

While Aucan and Bulel differed in their overall content, the qualitative profile of AR homologues was similar, with AR 21:0 being the most abundant followed by AR 23:0, AR 19:0, AR 25:0, AR 17:0, AR 21:1, and AR 19:1. This trend is in line with *T. turgidum* spp. *durum* from which tritordeum derives,^42^ confirming the relevance of genetic heritability of such a trait.

Conversely to the trend observed for common wheat varieties, the AR content in tritordeum varieties did not change significantly over the two harvesting years, suggesting a lower susceptibility to environmental conditions (*p* > 0.05).

As with wheat cultivars, the AR 17:0/AR 21:0 ratio for *Tritordeum* was calculated (Table 2) providing concentration values in the range of 0.057–0.078, suggesting the remarkable heritability of AR contents from tritordeum parents, namely, durum wheat (0.01–0.02) and barley (0.05–0.46).^45^

Also, the AR 21:0/AR 23:0 ratio was calculated showing the lowest value (1.50 ± 0.27) compared to the other species considered in the present study. This can be explained considering the ratio of this durum wheat parent,^14^ in which resistance to fungal ear infection is very low.^46^ Therefore, considering this indicator, tritordeum appears to be potentially a species susceptible to Fusarium head blight. This data is confirmed by a recent study since the mycotoxin contamination found in tritordeum samples was comparable to that of durum wheat, while it was higher compared to that of common wheat.^47^ These values may be considered also as a potential marker for authenticity, as suggested for other cereals.^42^

### Detection and Identification of 5-(2-Oxo)alkylresorcinols

Five 5-(2-oxo)AR were putatively identified, as summarized in Table 4, including the oxidized forms of AR 21:0, AR 21:1, AR 23:0, AR 23:1, and AR 25:0 (see Figure S2 of the Supporting Information). These derivatives were reported for the first time in rye, and their presence in all the evaluated samples suggests a higher diversity in the AR profile than previously suggested.^20,48^

**Table 4:** Alkylresorcinols Annotated in This Study^c^

compound	adduct	observed *m*/*z*	mass error (ppm)	molecular formula	RT (min)	fragmentation (*m*/*z*)	observed CCS (Å^2^)	RSD (%)
AR 21:1 oxo	[M – H]^−^	415.3218	–1.7	C_27_H_44_O_3_	5.57	123.0448; 81.0340	215.5	0.06
AR 17:0^a^	[M – H]^−^	347.2954	–2.5	C_23_H_40_O_2_	5.90	305.2844	204.6	0.05
AR 21:0 oxo	[M – H]^−^	417.3374	–2.3	C_27_H_46_O_3_	6.03	375.3255; 123.0446; 81.0340	218.1	0.003
AR 23:1 oxo	[M – H]^−^	443.3531	–0.9	C_29_H_48_O_3_	6.10	b	224.0	0.10
AR 19:1	[M + HCOO]^−^	419.3162	–2.4	C_25_H_42_O_2_	6.15	373.3101; 331.2995	219.6	0.76
AR 19:0^a^	[M – H]^−^	375.3269	–1.7	C_25_H_44_O_2_	6.52	333.3152	213.0	0.22
AR 21:1	[M + HCOO]^−^	447.3480	–2.9	C_27_H_46_O_2_	6.58	401.3414; 359.3308	229.3	0.47
AR 23:0 oxo	[M – H]^−^	445.3687	–2.4	C_29_H_50_O_3_	6.59	403.3569; 123.0446; 81.0340	226.4	0.02
AR 21:0^a^	[M – H]^−^	403.3581	–2.4	C_27_H_48_O_2_	7.01	361.3467	221.9	0.03
AR 25:0 oxo	[M – H]^−^	473.4000	–2.4	C_31_H_54_O_3_	7.14	431.3989; 123.0444	238.0	0.01
AR 23:0^a^	[M – H]^−^	431.3895	–2.8	C_29_H_52_O_2_	7.55	389.3782	230.4	0.01
AR 25:0	[M – H]^−^	459.4207	–1.6	C_31_H_56_O_2_	8.08	417.4090	244.5	0.20

a Confirmation with the standard by comparison of accurate mass, HRMS/MS, RT, and CCS.

b No HRMS/MS was recorded due to the low ion intensity.

c Details on their retention times, fragments, and CCS-TWIMS-derived values are included in the table.

Since analytical standards are not available, we tentatively identified them using HRMS checking the exact mass (mass error less than 3 ppm), the match of experimental and theoretical isotope pattern in terms of spacing and relative intensities, comparing their retention time with the corresponding nonoxidized homologue and by investigating their fragmentation pattern.

**Figure 3:**
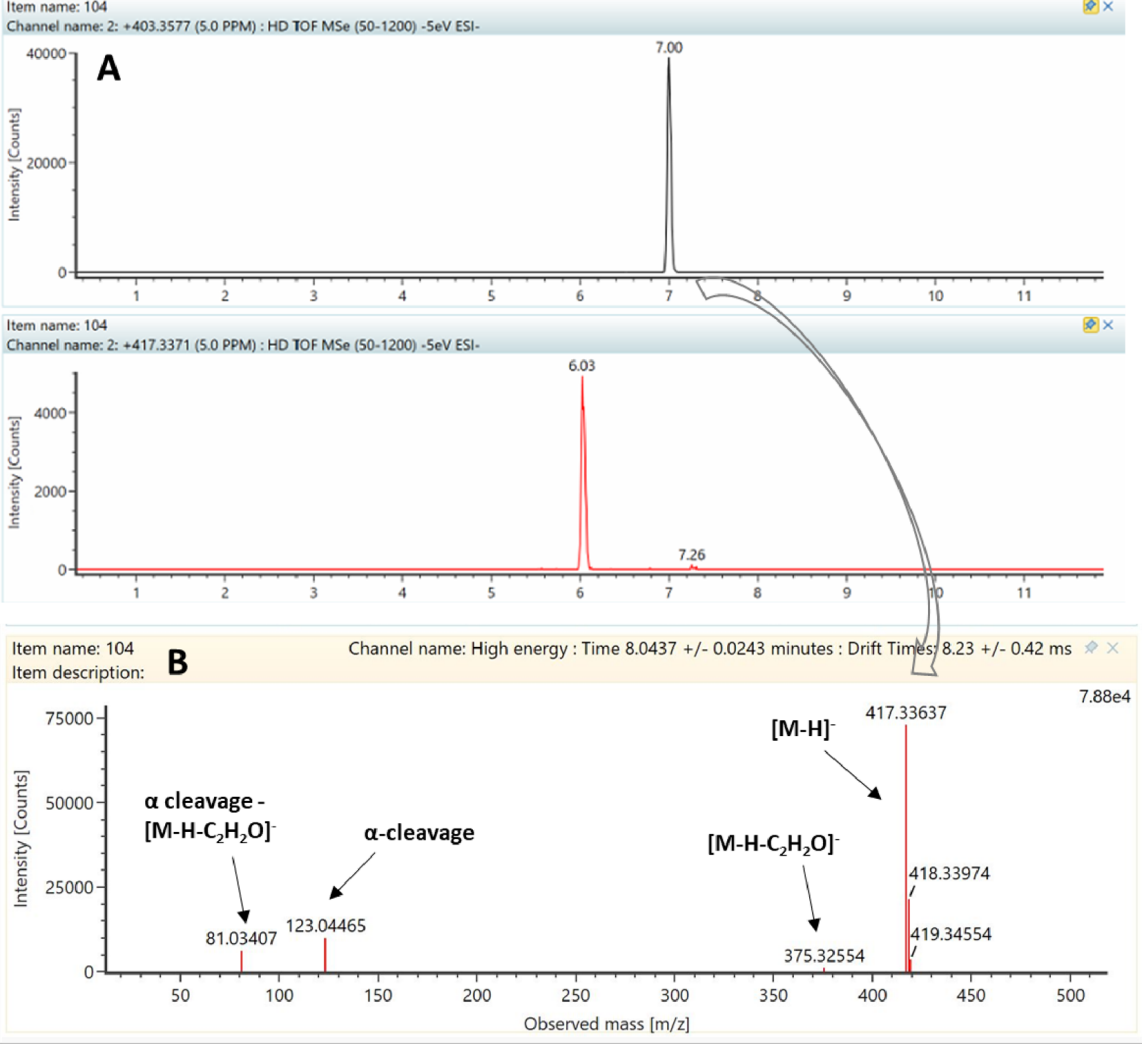
LC–HRMS/MS spectrum of the AR 21:0 oxo deprotonated adduct. (A) UPLC–Q-ToF full scan extracted ion chromatogram (extraction window of 5 ppm) of AR 21:0 [M – H]^−^*m*/*z* 403.3577 and AR 21:0 oxo [M – H]^−^*m*/*z* 417.3371 in wheat samples. (B) High-resolution fragmentation spectrum of AR 21:0 oxo, showing the characteristic fragment ions.

As an example, the AR 21:0 oxo was identified in the full scan mass spectrum at *m*/*z* 417.3364, corresponding to the [M – H]^−^ ion with a putative formula C_27_H_46_O_3_ (Figure 3). To verify the position of the oxidation on the alkyl chain, the fragmentation pattern was further considered. The high-energy fragmentation spectrum (Figure 3) shows the characteristic fragment ion [M – C_2_H_2_O]^−^ from the resorcinol ring, resulting from the neutral loss of 42 Da (*m*/*z* 375.3254). The other two fragment ions were identified corresponding to the α-cleavage (*m*/*z* 123.0446) and its rearrangement (*m*/*z* 81.0341). Indeed, as previously described,^20^ the cleavage arose from the α-position of carbonyl, suggesting that the oxidation occurred in position 2′. Furthermore, the earlier elution (6.03 min) of such a form from the C18 column compared with its nonoxidized homologue AR 21:0 (7.0 min) was consistent with the putative identification. The fragmentation mass spectra of the other AR oxo are show in the Supporting Information (Figure S3).

5-(2-Oxo)AR were found in rye and in other winter cereals as reported by Suzuki,^20,48^ but there was no information about their relative abundance in different cereal species. Different from saturated AR in which the most abundant homologue was AR 21:0, the most abundant oxo homologue was AR 23:0 oxo in all five species followed by AR 21:0 oxo, AR 25:0 oxo, AR 23:1 oxo, and AR 21:1 oxo in common wheat and spelt. On the other hand, for einkorn, emmer, and tritordeum, AR 23:0 oxo was followed by AR 25:0 oxo, AR 21:0 oxo, AR 23:1 oxo, and AR 21:1 oxo (see Figure S4 of the Supporting Information).

Einkorn and emmer showed the highest contents of all the 5-(2-oxo)AR homologues. This might be due to the accumulation of reactive oxygen species (ROS)^49^ caused by several biotic and abiotic stressors. Indeed, considering the ratio AR 21:0/AR 23:0 previously described, these two species can be considered more susceptible to fungal infestation, and thus, they may be exposed to higher oxidative stress.

Few publications reported that 5-(2-oxo)AR derives from a common intermediate of AR, which is a β fatty acid, and not directly from oxidation of AR.^20,50^ This could explain why the major 5-(2-oxo)AR homologue detected in the present study was AR 23:0 oxo, while the saturated one was AR 21:0. Indeed, it may be postulated that the lower accumulation of AR 23:0 was due to the preferred synthesis of the corresponding oxo homologue, resulting in a lower accumulation of saturated AR 23:0.

Further studies are required to understand the role in plant of these AR and the modulation of their biosynthetic route, but their widespread presence in different species and cultivar is confirmed by our data.

Furthermore, conjugated derivatives of AR were searched. The complete database of glucoside and methyl derivatives is reported in Table S4 of the Supporting Information. However, we could not find any of them. So far, conjugated derivatives were already reported for *Cybianthus magnus*(21) and *Grevillea robusta*.^22^ It should be mentioned that the glucose moiety is linked to a short-alkyl chain AR (2 to 6 carbon atoms), while in cereal AR, homologues from 15 C are present.

### Localization of AR in Common Wheat by Mass Spectrometry Imaging

**Figure 4:**
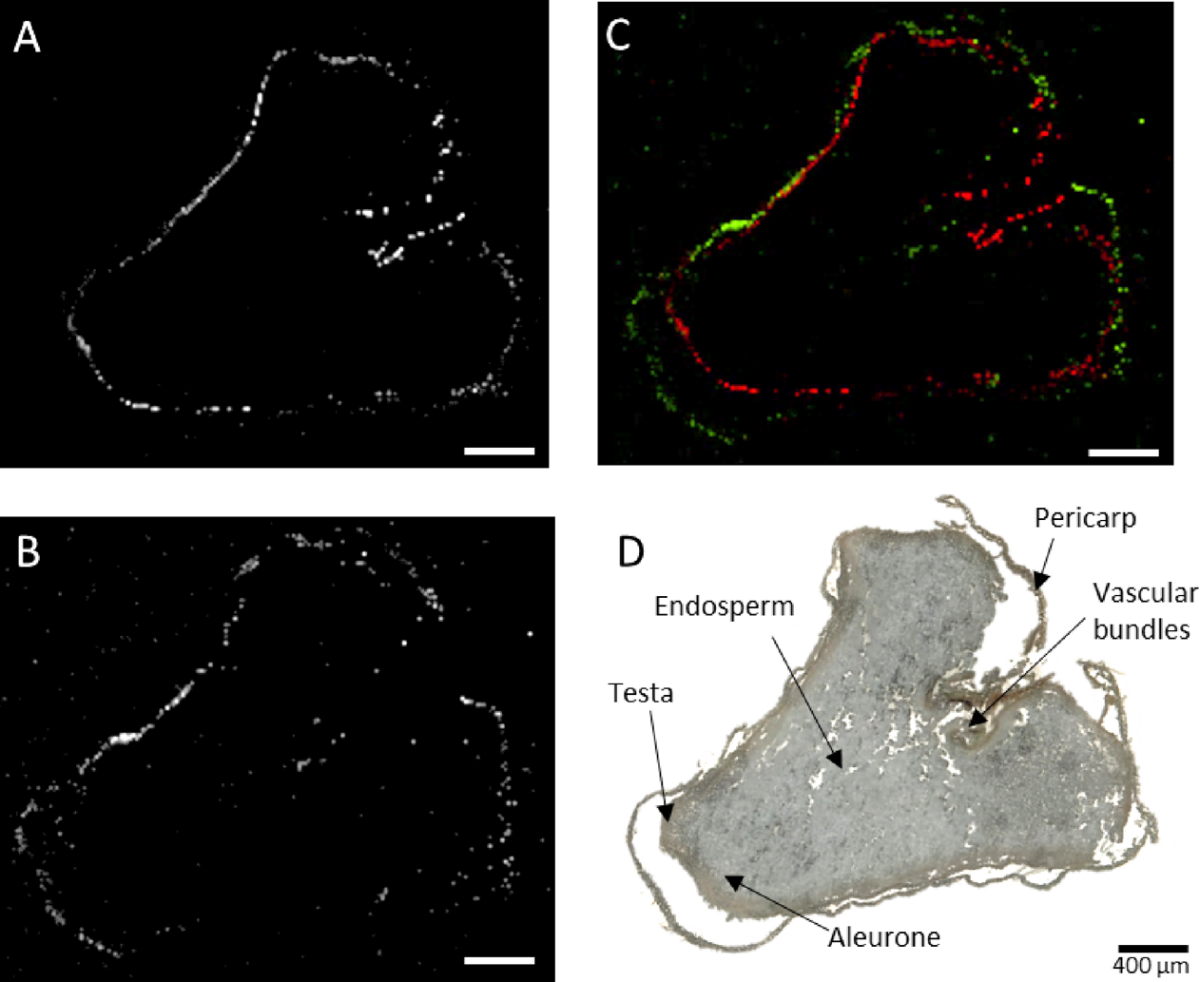
Alkylresorcinol spatial distributions in the cross section of a common wheat (*Triticum aestivum* spp. *aestivum*) kernel. (A) AR 21:0 [M + H]^+^*m*/*z* 405.3727 was found to be accumulated in the cuticle of the testa, while (B) AR 21:1 [M + Na]^+^*m*/*z* 425.3390 was mainly located in the outer cuticle of the pericarp. (C) Overlay image of AR 21:0 [M + H]^+^*m*/*z* 405.3727 (red) and AR 21:1 [M + Na]^+^*m*/*z* 425.3390 (green). (D) Optical image of the common wheat seed section with major morphological features labeled. MS images of common wheat kernel were generated with 183 × 149 pixels, 20 μm × 20 μm pixel size, and an *m*/*z* bin width of ±5 ppm. Scale bars: 400 μm.

AR were also investigated by MSI to unveil their spatial distribution within the common wheat kernel. Localizations of AR were studied using transversal cross sections made from the middle of the grain. Six AR were detected and localized at the tissue level, among other also unsaturated side-chain homologues, including AR 19:0, AR 19:1, AR 21:0, AR 21:1, AR 23:0, and AR 25:0. Their MSI images are reported in Figure 4 and in the Supporting Information (Figure S5).

Homologues ranging from C19 to C25 were detected as protonated and/or sodiated ion species, while AR 17:0 was not found. This might be due to its low concentration and to its lower ionization efficiency compared to the longer homologues.

A distinct pattern of accumulation was noticed for separate homologues. In particular, AR 21:0 was found to be accumulated mainly in the cuticle of the testa, while AR 21:1 was located both in the inner and outer cuticle of the pericarp (see Figure 4). Also, AR 19:0, AR 19:1, AR 23:0, and AR 25:0 were colocalized in the testa and the pericarp. Their spatial distributions are shown in the Supporting Information (Figure S5). No AR were found in the endosperm or in the germ layers.

AR distribution has been traditionally investigated by analyzing their content in pearling fractions, and microscopy has targeted them to the pericarp layer.^37,51^ Here, the exact location of different AR homologues has been made possible by the use of high-resolution AP-SMALDI MS imaging.

In winter cereals, these secondary metabolites are reported to inhibit the growth and spread of fungal infections;^6^ thus, their localization is consistent with their biological role as antifungal compounds. Indeed, their amphiphilic structure suggests their involvement in plant defense, and the concentration within the outer layers seems to be sufficient to act as a chemical barrier against the fungal spread. These data complement, with the accuracy of the details allowed by MS imaging, previous reports on the distribution of AR in wheat kernels.

## Supporting Information Available

(Table S1) Main in-house validation parameters; (Figure S1) AR composition of different winter wheat species; (Table S2) monthly cumulative rainfall and growing degree days; (Table S3) grain yield, test weight, and thousand-kernel weight on different cultivars of einkorn, emmer, spelt, common wheat, and tritordeum; (Figure S2) chemical structures of alkylresorcinols investigated in the present study; (Figure S3) HRMS/MS spectrum of 5-(2-oxo)AR putatively identified in the present study; (Figure S4) 5-(2-oxo)AR homologue relative abundance in different cereal species; (Table S4) alkylresorcinol database including the molecular formula, *m*/*z* values, and predicted CCS values (PDF)

## Notes

Mass spectrometry imaging data that support the findings of this study were deposited in the Metaspace database (https://metaspace2020.eu) with the accession code JLU Giessen_WS_inf_DON_183x149_20_A15.
