# Supplementary material for: 5-n-Alkylresorcinol Profiles in Different Cultivars of Einkorn, Emmer, Spelt, Common Wheat, and Tritordeum
Source: J Agric Food Chem. 2021 Nov 18;69(47):14092–102. doi: 10.1021/acs.jafc.1c05451 (PMC8640985; doi:10.1021/acs.jafc.1c05451)
Supplement: Supplementary file 1 — jf1c05451_si_001.pdf [file jf1c05451_si_001.pdf]

Supporting Information for:

5-n-Alkylresorcinols profile in different cultivars of einkorn, emmer, spelt, common wheat and tritordeum.

Clara Pedrazzani[1], Francesca Vanara[2], Dhaka Ram Bhandari[3], Renato Bruni[1], Bernhard Spengler[3], Massimo Blandino[2]*, Laura Righetti[1]*

[1] *Department of Food and Drug, University of Parma, Parco Area delle Scienze 17/A, 43124 Parma, Italy*

[2] *Department of Agricultural, Forest and Food Sciences, University of Torino, Largo Paolo Braccini, 2, Grugliasco, Italy*

[3] *Institute of Inorganic and Analytical Chemistry, Justus Liebig University Giessen, Heinrich-Buff-Ring 17, 35392 Giessen, Germany.*

* Corresponding author:

Dr. Laura Righetti: laura.righetti@unipr.it

Prof. Massimo Blandino: massimo.blandino@unito.it

Agronomic management of field experiments

The same agronomic technique was adopted for all cultivars of common wheat, einkorn, emmer, spelt and tritordeum.

In both years the previous crop was maize in Cigliano and common wheat in Carmagnola. Planting was performed in the end of October in 12 cm wide rows at a seeding rate of 300 seeds m^{-2} for einkorn, emmer and spelt and 400 seeds m^{-2} for common wheat and tritordeum, following an autumn plowing (30 cm) and disk harrowing to prepare a proper seedbed. Each experimental field received 50 kg ha^{-1} of P_2O_5 and 66 kg ha^{-1} K_2O each year after plowing. For all cultivars, 80 kg of nitrogen ha^{-1} were applied as a granular ammonium nitrate fertilizer, split equally at tillering (growth stage 23) and at the beginning of stem elongation (growth stage 32). The weed control was conducted with Fluroxipir, Clopiralid and MCPA (Manta Gold, Syngenta Italia, Milan, Italy) and Pinoxaden (Axial Pronto, Syngenta Italia,) at wheat tillering, while no fungicide and insecticide have been applied during cultivation. Harvesting was carried out with a combine-harvester in the beginning of July. Treatments were assigned to experimental units using a completely randomised block design with three replicates.

Table 1. Main in-house validation parameters.

	AR 17:0	AR 19:0	AR21:0	AR 23:0
Calibration range (mg/kg)[a]	0.5-25	0.1-25	0.1-25	0.1-25
LCL (mg/kg)[b]	0.1	0.03	0.02	0.02
LOQ (mg/kg)	0.5	0.1	0.1	0.1

[a] High linearity (R^2>0.99) has been observed in the used calibration.
[b] LCL: lowest calibration level [1]

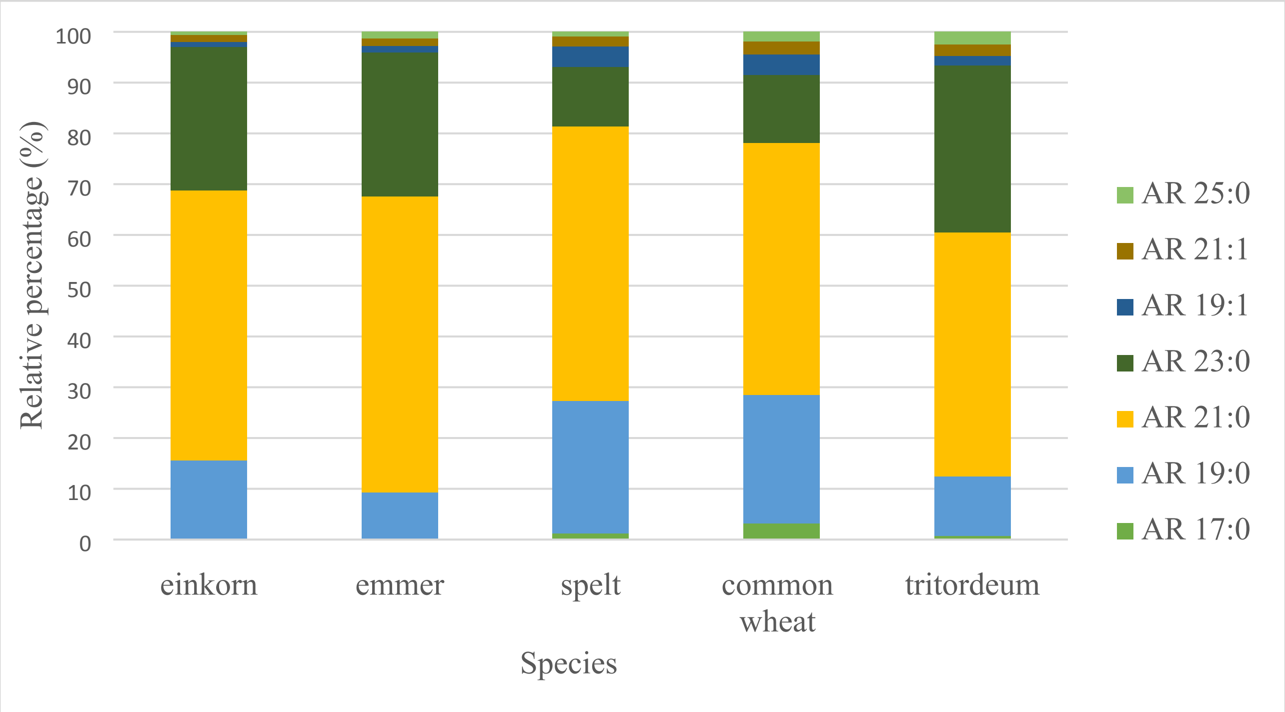

Figure 1. AR composition, expressed as relative percentages of AR homologues, of different winter wheat species.

Table 2. Monthly cumulative rainfall and growing degree days (GDDs)[1] measured in the experimental areas from sowing (November) to the end of ripening (June).

Year	Month	Rainfall (mm) [1]		GDDs (Σ °C-day)[2]	
		Cigliano	Carmagnola	Cigliano	Carmagnola
2016-17	November	158	257	238	250
	December	45	77	144	159
	January	4	12	97	111
	February	45	62	152	175
	March	69	69	349	356
	April	34	51	415	412
	May	79	77	554	558
	June	149	103	673	698
	November - June	582	708	2622	2718
	April - May	114	128	970	970
2017-18	November	48	66	224	220
	December	33	27	113	82
	January	107	117	178	141
	February	60	86	113	89
	March	109	103	223	209
	April	93	116	456	444
	May	138	310	583	565
	June	35	14	665	672
	November - June	622	837	2554	2421
	April - May	230	425	1039	1009

[1] Data obtained from the Regione Piemonte agrometeorological service.

[2] Accumulated growing degree days for each experiment using a 0°C base value.

Table 3. Grain yield, test weight (TW) and thousand-kernel weight (TKW) on different cultivars of einkorn, emmer, spelt, common wheat and tritordeum cultivated in 2 growing seasons.

		Grain yield (t ha^{-1})		TW (kg hl^{-1})		TKW (g)	
**Species**	**Cultivar**	**2017**	**2018**	**2017**	**2018**	**2017**	**2018**
einkorn	Monlis	2.1 j	1.0 i	71.7 def	66.6 d	31.0 k	25.7 h
emmer	Luni	3.2 ghij	3.3 de	64.0 i	57.5 g	48.4 def	43.6 c
	Giovanni Paolo	4.2 def	2.1 h	67.0 g	60.0 f	56.7 a	43.2 c
spelt	BC Vigor	4.5 cde	4.1 bc	65.1 hi	62.5 e	48.6 cde	49.5 a
	Rossella	4.1 defg	4.4 ab	65.9 gh	62.5 ef	58.8 a	48.0 ab
common wheat	Andriolo	2.7 hij	2.1 gh	74.9 bc	73.5 abc	46.0 f	42.3 c
	Gentilrosso	2.7 ij	2.8 efg	75.8 bc	74.6 ab	53.9 c	46.5 b
	Frassineto	2.5 j	2.4 fgh	74.6 bc	74.2 ab	51.1 b	47.8 ab
	Verna	2.9 hij	2.8 ef	75.9 b	74.7 ab	42.3 gh	43.2 c
	Bologna	5.7 ab	4.9 ab	78.3 a	76.1 a	34.5 j	31.4 ef
	Aubusson	6.1 ab	4.5 ab	73.1 d	66.8 d	40.6 ghi	32.4 e
	Solehio	6.5 a	5.0 a	75.3 bc	72.4 bc	47.0 def	43.1 c
	Arabia	6.4 a	4.9 ab	75.3 bc	71.0 c	46.9 ef	38.5 d
	Bonavita	4.2 def	3.2 de	74.5 c	66.6 d	38.7 i	28.9 g
	Rosso	4.7 cd	3.7 cd	72.2 de	71.3 c	42.5 g	37.4 d
	Skorpion	5.3 bc	3.2 de	71.3 ef	63.5 e	49.7 cd	38.7 d
tritordeum	Aucan	3.6 efgh	2.0 h	70.6 f	55.6 g	38.9 i	27.3 gh
	Bulel	3.6 fghi	3.0 def	71.9 def	60.6 ef	40.2 hi	29.6 fg

Data are average of 2 sites in North West Italy

Means of species followed by different letters are significantly different ($p < 0.001$), according to the Tukey's post hoc test.

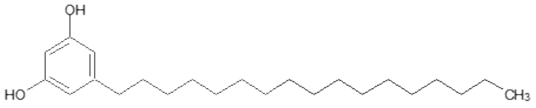

AR 17:0

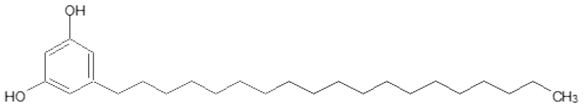

AR 19:0

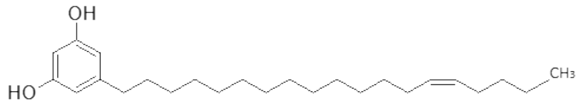

AR 19:1

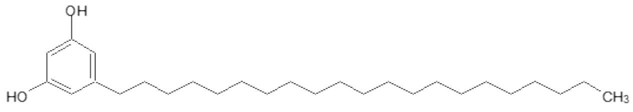

AR 21:0

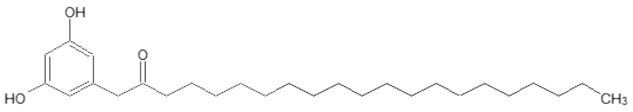

AR 21:0 oxo

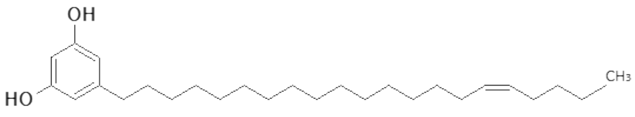

AR 21:1

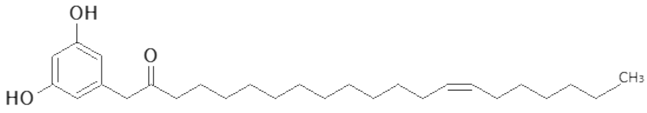

AR 21:1 oxo

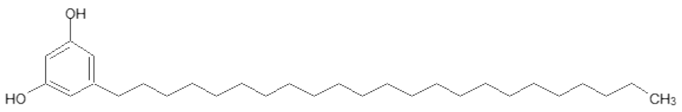

AR 23:0

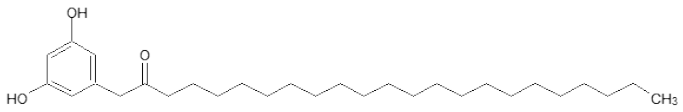

AR 23:0 oxo

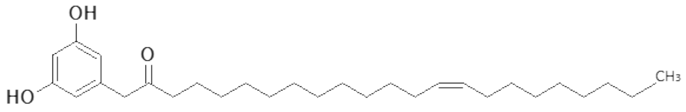

AR 23:1 oxo

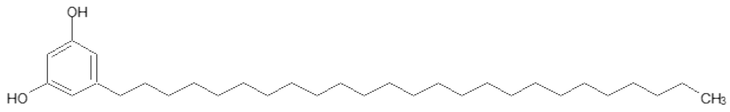

AR 25:0

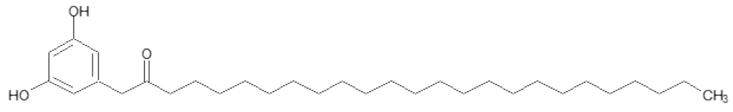

AR 25:0 oxo

Figure 2. Chemical structures of alkylresorcinols investigated in the present study.

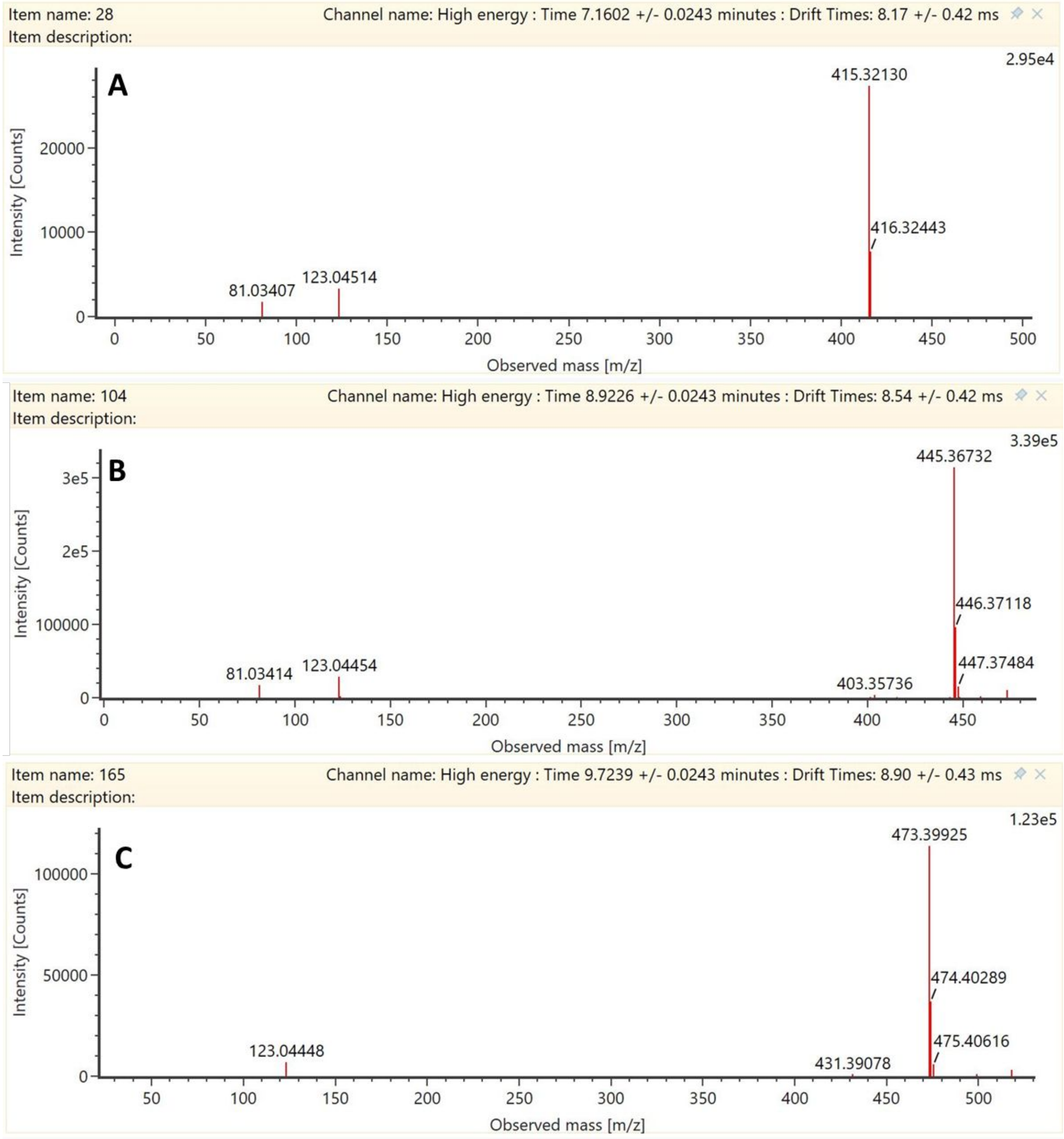

Figure 3. HRMS/MS spectrum of 5-(2-oxo) AR putatively identified in the present study. High-resolution fragmentation spectrum of deprotonated (A) AR 21:1 oxo, (B) AR 23:0 oxo, and (C) AR 25:0 oxo.

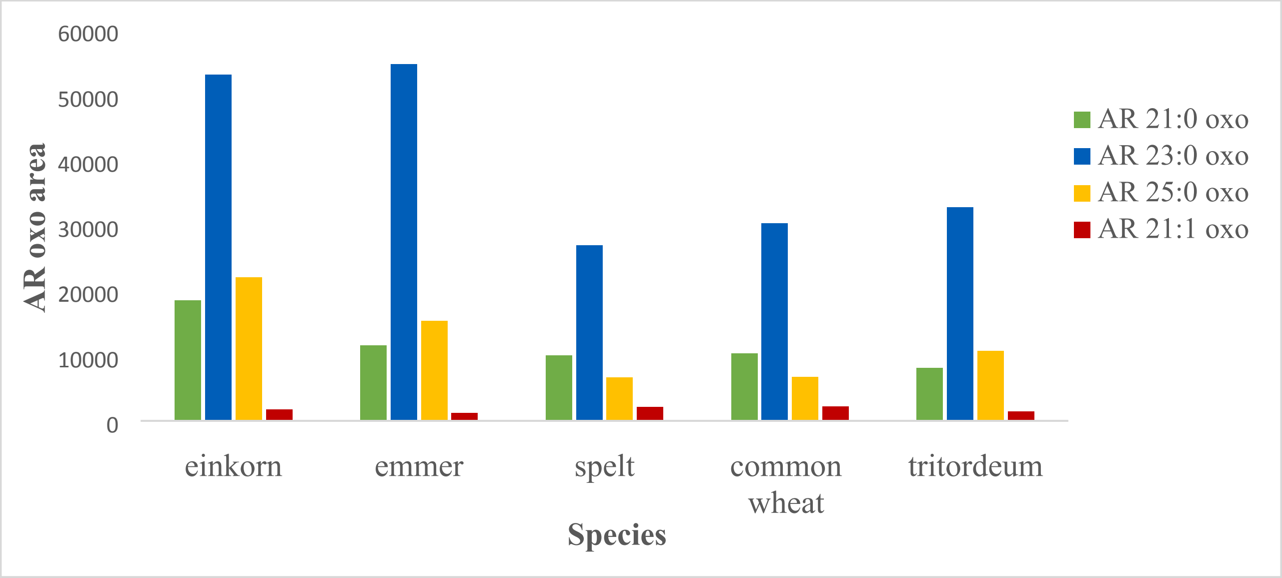

Figure 4. 5-(2-oxo) AR homologue relative abundance in different cereal species.

Table 4. Alkylresorcinol database including molecular formula, *m/z* values, predicted CCS values.

Compound	Molecular formula	Theoretical CCS* $[M-H]^-$	Theoretical CCS* $[M+HCOO]^-$	Theoretical CCS* $[M-H]^-$
		Predicted with AllCCS		Predicted with CCSBase
AR 17:0 glucoside	$C_{29}H_{50}O_7$	223.1	229.5	233.5
AR 19:0 glucoside	$C_{31}H_{54}O_7$	229.5	237.0	242.0
AR 21:0 glucoside	$C_{33}H_{58}O_7$	235.7	244.2	250.5
AR 23:0 glucoside	$C_{35}H_{62}O_7$	241.6	251.0	258.9
AR 17:0 2'OAR	$C_{23}H_{38}O_3$	195.7	199.6	195.9
AR 17:1 2'OAR	$C_{23}H_{36}O_3$	194.3	198.3	194.9
AR 17:2 2'OAR	$C_{23}H_{34}O_3$	193.5	197.2	193.9
AR 19:0 2'OAR	$C_{25}H_{42}O_3$	202.5	207.5	204.7
AR 19:1 2'OAR	$C_{25}H_{40}O_3$	201.2	206.4	203.6
AR 19:2 2'OAR	$C_{25}H_{38}O_3$	200.5	205.5	202.6
AR 21:2 2'OAR	$C_{27}H_{42}O_3$	207.6	213.9	211.2
AR 23:2 2'OAR	$C_{29}H_{46}O_3$	214.9	222.5	219.8
AR 25:1 2'OAR	$C_{31}H_{52}O_3$	222.2	231.1	229.5
AR 25:2 2'OAR	$C_{31}H_{50}O_3$	222.5	231.4	228.4
AR 27:0 2'OAR	$C_{33}H_{58}O_3$	227.3	236.5	239.2
AR 27:1 2'OAR	$C_{33}H_{56}O_3$	229.3	239.3	238.0
AR 27:2 2'OAR	$C_{33}H_{54}O_3$	230.3	240.5	236.9
AR 17:0 bcARa	$C_{23}H_{40}O_2$	192.5	196.7	194.7
AR 19:0 bcARa	$C_{25}H_{44}O_2$	199.1	204.3	203.6
AR 21:0 bcARa	$C_{27}H_{48}O_2$	205.5	211.8	212.3
AR 21:0 bcARb	$C_{27}H_{48}O_3$	205.8	212.0	212.3
AR C23:0 bcARa	$C_{29}H_{52}O_2$	211.9	219.4	221.0
AR C23:0 bcARb	$C_{29}H_{52}O_3$	212.2	219.5	221.0
AR 25:0 bcARa	$C_{31}H_{56}O_2$	218.2	226.9	229.7
AR 17:0 mAR	$C_{24}H_{42}O_2$	196.3	200.9	199.0
AR 19:0 mAR	$C_{26}H_{46}O_2$	202.9	208.5	207.8
AR 21:0 mAR	$C_{28}H_{50}O_2$	209.3	216.0	216.5
AR 23:0 mAR	$C_{30}H_{54}O_2$	215.6	223.4	225.3
AR 25:0 mAR	$C_{32}H_{58}O_2$	221.8	230.7	233.9
grevilloside A	$C_{17}H_{24}O_8$	180.2	181.1	182.5
grevilloside B	$C_{17}H_{26}O_8$	183.2	182.8	183.5
grevilloside C	$C_{17}H_{24}O_9$	182.9	183.7	183.9
grevilloside D	$C_{16}H_{24}O_8$	178.3	178.9	179.1
grevilloside E	$C_{16}H_{22}O_9$	179.3	179.9	179.6
grevilloside F	$C_{15}H_{18}O_9$	174.5	174.6	174.2
grevilloside G	$C_{14}H_{20}O_8$	170.5	170.7	170.4
grevilloside H	$C_{18}H_{28}O_8$	185.4	186.5	187.8
grevilloside E methyl ester	$C_{17}H_{24}O_9$	183.6	184.6	184.1
grevilloside I	$C_{17}H_{24}O_{10}$	185.3	186.1	185.8

* Theoretical CCS were obtained with a model trained with machine learning approaches proposed by Ross et al.[2] CCSbase (https://ccsbase.net/) and by Zhou et al. [3] namely AllCCS (http://allccs.zhulab.cn/ . In brief, using a training set of experimentally measured CCS, the software employs a machine learning algorithm able to predict CCS values for novel structures. To calculate the predicted CCS for $[M-HCOO]^-$ and $[M-H]^-$ adducts, the SMILES string of each AR were drawn and imported into CCSbase.

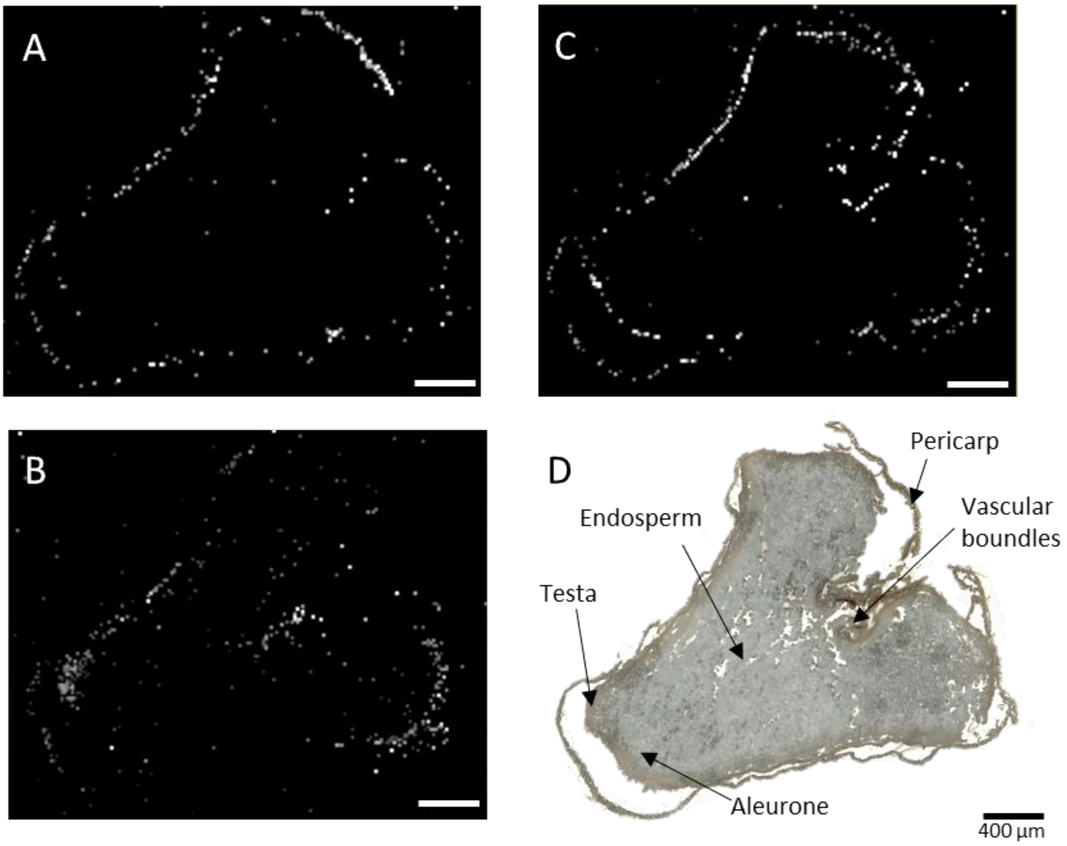

Figure 5. Alkylresorcinols spatial distribution in the cross section of a common wheat *(Triticum aestivum spp aestivum)* kernel.
(A) AR 25:O $[M+H]^+$ *m/z* 461.4353, (B) AR 19:0 $[M+Na]^+$ *m/z* 399.3233 and (C) AR 23:0 $[M+Na]^+$ *m/z* 433.4040 were found to be accumulated in the cuticle of the testa and in the outer cuticle of the pericarp.
(D) Optical image of a common wheat seed section with major morphological features labeled.
MS images of infected kernel were generated with 183 x 149 pixels; 20 µm x 20 µm pixel size; *m/z* bin width: ±5 ppm. Scale bars: 400 µm.

References

1. Zachariasova, M.; Cajka, T.; Godula, M.; Malachova, A.; Veprikova, Z.; Hajslova, J. Analysis of multiple mycotoxins in beer employing (ultra)-high-resolution mass spectrometry. *Rapid Commun. Mass Spectrom.* **2010**, *24*, doi:10.1002/rcm.4746.
2. Ross, D.H.; Cho, J.H.; Xu, L. Breaking Down Structural Diversity for Comprehensive Prediction of Ion-Neutral Collision Cross Sections. *Anal. Chem.* **2020**, *92*, 4548–4557, doi:10.1021/acs.analchem.9b05772.
3. Zhou, Z.; Shen, X.; Tu, J.; Zhu, Z.J. Large-scale prediction of collision cross-section values for metabolites in ion mobility-mass spectrometry. *Anal. Chem.* **2016**, *88*, 11084–11091, doi:10.1021/acs.analchem.6b03091.
